# 3-(2-Bromo­acet­yl)phenyl benzoate

**DOI:** 10.1107/S1600536813002900

**Published:** 2013-02-02

**Authors:** Sachin P Ambekar, H. C. Devarajegowda, J. ShylajaKumari, K. Mahesh Kumar, O. Kotresh

**Affiliations:** a Department of Chemistry, Karnatak University’s Karnatak Science College, Dharwad, Karnataka 580 001, India; b Department of Physics, Yuvaraja’s College (Constituent College), University of Mysore, Mysore 570 005, Karnataka, India; c Department of Physics, AVK College for Women, Hassan 573 201, Karnataka, India

## Abstract

In the title compound, C_15_H_11_BrO_3_, the dihedral angle between the benzene rings is 72.59 (6)°. In the crystal, pairs of C—H⋯π contacts form inversion dimers. Additional C—H⋯O hydrogen bonds generate *R*_2_^1^(6) ring motifs and stack these dimers along the *b* axis. Short inter­molecular Br⋯O contacts of 3.254 (3) Å are also observed and link the stacks into a three-dimensional network.

## Related literature

For the biological applications and synthesis of the title compound, see: Naoto *et al.* (2008 ▶); Shwu-Jiuan & Mei-Hua (1984 ▶); Jaakko & Erkki (1959 ▶); Junichi *et al.* (1956 ▶); D’Amico *et al.* (1956 ▶). For hydrogen-bond motifs, see: Bernstein *et al.* (1995 ▶)
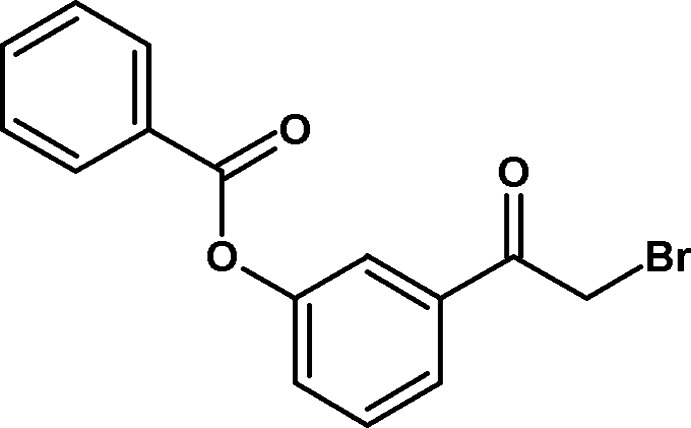


## Experimental

### 

#### Crystal data

C_15_H_11_BrO_3_
*M* *_r_* = 319.15
Monoclinic, 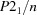

*a* = 12.5055 (4) Å
*b* = 5.4409 (2) Å
*c* = 19.5178 (6) Å
β = 90.859 (2)°
*V* = 1327.86 (8) Å^3^
*Z* = 4
Mo *K*α radiation
μ = 3.10 mm^−1^
*T* = 296 K
0.24 × 0.20 × 0.12 mm

#### Data collection

Bruker SMART CCD area-detector diffractometer
Absorption correction: multi-scan (*SADABS*; Sheldrick, 2007 ▶) *T*_min_ = 0.770, *T*_max_ = 1.000
11209 measured reflections
2344 independent reflections
1929 reflections with *I* > 2σ(*I*)
*R*_int_ = 0.033

#### Refinement

*R*[*F* ^2^ > 2σ(*F* ^2^)] = 0.033
*wR*(*F* ^2^) = 0.092
*S* = 1.05
2344 reflections
172 parameters
H-atom parameters constrained
Δρ_max_ = 0.37 e Å^−3^
Δρ_min_ = −0.62 e Å^−3^

### 

Data collection: *SMART* (Bruker, 2001 ▶); cell refinement: *SAINT* (Bruker, 2001 ▶); data reduction: *SAINT*; program(s) used to solve structure: *SHELXS97* (Sheldrick, 2008 ▶); program(s) used to refine structure: *SHELXL97* (Sheldrick, 2008 ▶); molecular graphics: *ORTEP-3 for Windows* (Farrugia, 2012 ▶); software used to prepare material for publication: *SHELXL97*.

## Supplementary Material

Crystal structure: contains datablock(s) I, global. DOI: 10.1107/S1600536813002900/sj5298sup1.cif

Structure factors: contains datablock(s) I. DOI: 10.1107/S1600536813002900/sj5298Isup2.hkl

Supplementary material file. DOI: 10.1107/S1600536813002900/sj5298Isup3.cml

Additional supplementary materials:  crystallographic information; 3D view; checkCIF report

## Figures and Tables

**Table 1:** Hydrogen-bond geometry (Å, °) *Cg*2 is the centroid of the C10–C15 benzene ring.

*D*—H⋯*A*	*D*—H	H⋯*A*	*D*⋯*A*	*D*—H⋯*A*
C4—H4⋯O3^i^	0.93	2.70	3.557 (4)	153
C1—H1*B*⋯O3^i^	0.97	2.65	3.550 (4)	155
C6—H6⋯*Cg*2^ii^	0.93	2.88	3.627 (3)	138

Symmetry codes: (i) 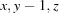; (ii) 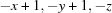.

**Fig. 1.**
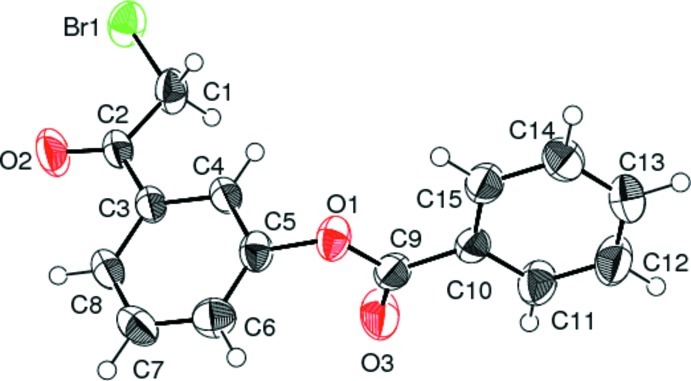
The molecular structure of the title compound. Displacement ellipsoids are drawn at the 50% probability level. Hydrogen atoms are shown as spheres of arbitrary radius.

**Fig. 2.**
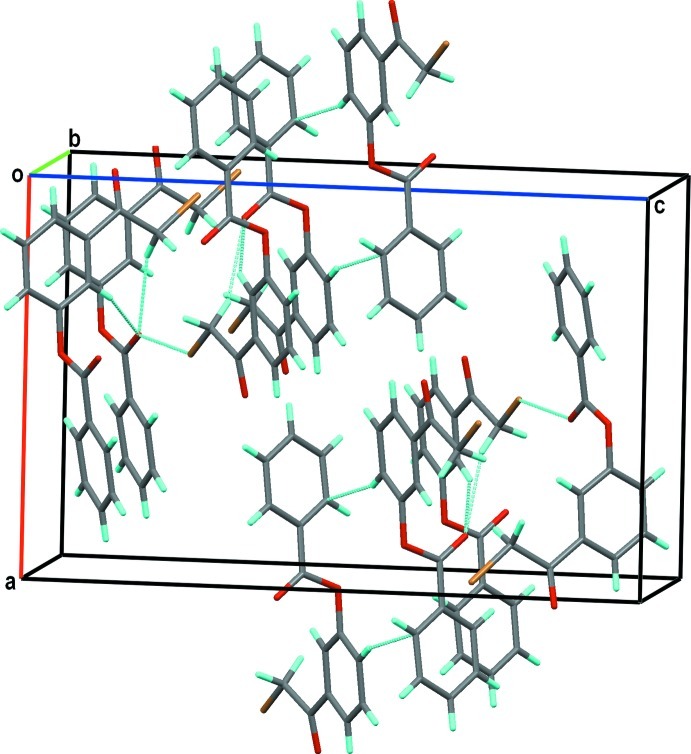
Crystal packing along b axis for the title compound with hydrogen bonds drawn as dashed lines.

## supplementary crystallographic information

### Comment

3-(2-bromoacetyl)phenyl benzoate is key starting material for the synthesis of phenylephrine, (*R*)-3-[-1-hydroxy-2-(methylamino)ethyl]phenol. Phenylephrine is a selective α_1_-adrenergic receptor agonist used primarily as a decongestant, as an agent to dilate the pupils, and to increase blood pressure.

Oral phenylephrine is extensively metabolized by monoamine oxidase (Naoto *et al.*, 2008), an enzyme that is present in the gastrointestinal tract and in the liver. Therefore, compared to intravenous pseudoephedrine, it has a reduced and variable bioavailability, only up to 38% (Shwu-Jiuan & Mei-Hua, 1984; Jaakko & Erkki, 1959). Because phenylephrine is a directly selective α-adrenergic receptor agonist, it does not cause the release of endogenous noradrenaline, as pseudoephedrine does. Therefore, phenylephrine is less likely to cause side effects such as central nervous system stimulation, insomnia, anxiety, irritability, and restlessness (Junichi *et al.*, 1956). Phenylephrine's effectiveness as a decongestant stems from its vasoconstriction of nasal blood vessels, thereby decreasing blood flow to the sinusoidal vessels, leading to decreased mucosal edema (D'Amico *et al.*, 1956).

The asymmetric unit of 3-(2-bromoacetylphenyl benzoate is shown in Fig. 1. The dihedral angle between two (C3–C8) and (C10—C15) benzene rings is 72.59 (6)°. In the crystal structure a pair of C6—H6···π [C_g_(2)(C10–C15) contacts form inversion dimers. Additional C4—H4···O3 and C1—H1B···O3 (Table.1) hydrogen bonds generate *R*^1^_2_(6) ring motifs (Bernstein *et al.*, 1995) and stack these dimers along the *b* axis (Fig. 2). Short intermolecular Br1···O2 contacts, 3.254 (3) Å are also observed and link the stacks into a three dimensional network.

### Experimental

All the chemicals used were of analytical reagent grade and were used directly without further purification. The title compound was synthesized according to an already reported method (Shwu-Jiuan & Mei-Hua, 1984). The crude product was recrystallized from an ethanol/chloroform mixture, to give colourless crystals in 78% yield.

### Refinement

All H atoms were positioned geometrically, with C—H = 0.93 Å for aromatic H and C—H = 0.97 Å for methylene H and refined using a riding model with *U*_iso_(H) = 1.2*U*_eq_(C) for aromatic and methylene H.

### Crystal data

**Table:**

C_15_H_11_BrO_3_	*F*(000) = 640
*M**_r_* = 319.15	*D*_x_ = 1.596 Mg m^−^^3^
Monoclinic, *P*2_1_/*n*	Melting point: 378 K
Hall symbol: -P 2yn	Mo *K*α radiation, λ = 0.71073 Å
*a* = 12.5055 (4) Å	Cell parameters from 2344 reflections
*b* = 5.4409 (2) Å	θ = 1.9–25.0°
*c* = 19.5178 (6) Å	µ = 3.10 mm^−^^1^
β = 90.859 (2)°	*T* = 296 K
*V* = 1327.86 (8) Å^3^	Plate, colourless
*Z* = 4	0.24 × 0.20 × 0.12 mm

### Data collection

**Table:**

Bruker SMART CCD area-detector diffractometer	2344 independent reflections
Radiation source: fine-focus sealed tube	1929 reflections with *I* > 2σ(*I*)
Graphite monochromator	*R*_int_ = 0.033
ω and φ scans	θ_max_ = 25.0°, θ_min_ = 1.9°
Absorption correction: multi-scan (*SADABS*; Sheldrick, 2007)	*h* = −14→14
*T*_min_ = 0.770, *T*_max_ = 1.000	*k* = −6→6
11209 measured reflections	*l* = −23→23

### Refinement

**Table:**

Refinement on *F*^2^	Primary atom site location: structure-invariant direct methods
Least-squares matrix: full	Secondary atom site location: difference Fourier map
*R*[*F*^2^ > 2σ(*F*^2^)] = 0.033	Hydrogen site location: inferred from neighbouring sites
*wR*(*F*^2^) = 0.092	H-atom parameters constrained
*S* = 1.05	*w* = 1/[σ^2^(*F*_o_^2^) + (0.0489*P*)^2^ + 0.8292*P*] where *P* = (*F*_o_^2^ + 2*F*_c_^2^)/3
2344 reflections	(Δ/σ)_max_ = 0.001
172 parameters	Δρ_max_ = 0.37 e Å^−^^3^
0 restraints	Δρ_min_ = −0.62 e Å^−^^3^

### Special details

**Table:**

Experimental. 3-(Bromo acetyl) phenyl benzoate: it was obtained as an off-white solid; M.P: 378k; GCMS data m/e 320 1H NMR (300 MHz, CDCl3, δ, p.p.m.): 4.46 (s 2H, Methylene-CH_2_),7.50 (s,1*H*, Ar—H), 7.52 (s,1*H*, Ar—H), 7.66 (s,1*H*, Ar—H), 7.68 (s,1*H*, Ar—H), 7.88 (s,1*H*, Ar—H), 7.90 (s,1*H*, Ar—H), 8.19 (d,2*H*, Ar—H),
Geometry. All s.u.'s (except the s.u. in the dihedral angle between two l.s. planes) are estimated using the full covariance matrix. The cell s.u.'s are taken into account individually in the estimation of s.u.'s in distances, angles and torsion angles; correlations between s.u.'s in cell parameters are only used when they are defined by crystal symmetry. An approximate (isotropic) treatment of cell s.u.'s is used for estimating s.u.'s involving l.s. planes.
Refinement. Refinement of *F*^2^ against ALL reflections. The weighted *R*-factor *wR* and goodness of fit *S* are based on *F*^2^, conventional *R*-factors *R* are based on *F*, with *F* set to zero for negative *F*^2^. The threshold expression of *F*^2^ > 2σ(*F*^2^) is used only for calculating *R*-factors(gt) *etc*. and is not relevant to the choice of reflections for refinement. *R*-factors based on *F*^2^ are statistically about twice as large as those based on *F*, and *R*- factors based on ALL data will be even larger.

### Fractional atomic coordinates and isotropic or equivalent isotropic displacement parameters (Å^2^)

**Table:**

	*x*	*y*	*z*	*U*_iso_*/*U*_eq_	
Br1	0.93093 (3)	−0.55127 (7)	0.763352 (16)	0.05813 (16)	
O1	0.57395 (15)	0.2166 (4)	0.92902 (10)	0.0458 (5)	
O2	1.00505 (17)	−0.2020 (5)	0.87494 (14)	0.0697 (7)	
O3	0.5845 (2)	0.5101 (5)	0.84970 (16)	0.0724 (8)	
C1	0.8494 (2)	−0.3090 (7)	0.81046 (16)	0.0524 (8)	
H1A	0.8210	−0.1928	0.7772	0.063*	
H1B	0.7893	−0.3891	0.8319	0.063*	
C2	0.9115 (2)	−0.1696 (6)	0.86438 (15)	0.0410 (7)	
C3	0.8497 (2)	0.0154 (5)	0.90374 (14)	0.0371 (7)	
C4	0.7386 (2)	0.0330 (6)	0.89847 (14)	0.0378 (6)	
H4	0.7002	−0.0766	0.8710	0.045*	
C5	0.6866 (2)	0.2118 (6)	0.93378 (14)	0.0396 (7)	
C6	0.7396 (3)	0.3758 (6)	0.97553 (16)	0.0489 (8)	
H6	0.7025	0.4965	0.9991	0.059*	
C7	0.8502 (3)	0.3567 (7)	0.98166 (16)	0.0519 (8)	
H7	0.8878	0.4647	1.0100	0.062*	
C8	0.9040 (2)	0.1796 (6)	0.94613 (14)	0.0438 (7)	
H8	0.9780	0.1693	0.9505	0.053*	
C9	0.5314 (2)	0.3695 (6)	0.88120 (16)	0.0430 (7)	
C10	0.4140 (2)	0.3355 (6)	0.87324 (15)	0.0392 (7)	
C11	0.3575 (3)	0.5030 (6)	0.83355 (19)	0.0564 (9)	
H11	0.3929	0.6327	0.8127	0.068*	
C12	0.2485 (3)	0.4773 (7)	0.8249 (2)	0.0626 (10)	
H12	0.2102	0.5922	0.7992	0.075*	
C13	0.1965 (3)	0.2843 (7)	0.85389 (17)	0.0553 (9)	
H13	0.1231	0.2664	0.8473	0.066*	
C14	0.2517 (3)	0.1179 (7)	0.89246 (18)	0.0546 (9)	
H14	0.2158	−0.0137	0.9120	0.066*	
C15	0.3611 (2)	0.1427 (6)	0.90285 (16)	0.0476 (7)	
H15	0.3984	0.0294	0.9297	0.057*	

### Atomic displacement parameters (Å^2^)

**Table:**

	*U*^11^	*U*^22^	*U*^33^	*U*^12^	*U*^13^	*U*^23^
Br1	0.0559 (2)	0.0682 (3)	0.0502 (2)	0.01670 (17)	−0.00215 (15)	−0.00925 (17)
O1	0.0314 (10)	0.0549 (13)	0.0512 (12)	0.0049 (10)	0.0054 (9)	0.0110 (10)
O2	0.0332 (13)	0.0820 (19)	0.0935 (18)	0.0101 (12)	−0.0161 (11)	−0.0277 (15)
O3	0.0443 (13)	0.0693 (17)	0.103 (2)	−0.0134 (12)	−0.0088 (13)	0.0430 (16)
C1	0.0366 (16)	0.069 (2)	0.0517 (17)	0.0138 (16)	−0.0050 (13)	−0.0151 (17)
C2	0.0282 (15)	0.0483 (18)	0.0463 (16)	−0.0014 (13)	−0.0045 (12)	0.0000 (15)
C3	0.0308 (14)	0.0420 (17)	0.0384 (15)	−0.0019 (12)	−0.0043 (12)	0.0032 (12)
C4	0.0319 (14)	0.0423 (16)	0.0391 (15)	−0.0034 (13)	−0.0045 (12)	0.0002 (13)
C5	0.0318 (14)	0.0472 (18)	0.0400 (15)	0.0001 (13)	0.0014 (12)	0.0079 (14)
C6	0.055 (2)	0.0480 (18)	0.0440 (17)	0.0006 (16)	0.0034 (14)	−0.0049 (15)
C7	0.054 (2)	0.055 (2)	0.0466 (17)	−0.0136 (17)	−0.0107 (14)	−0.0067 (16)
C8	0.0349 (15)	0.0523 (19)	0.0440 (16)	−0.0076 (14)	−0.0085 (13)	0.0005 (15)
C9	0.0398 (16)	0.0384 (16)	0.0510 (17)	0.0032 (14)	0.0036 (14)	0.0030 (15)
C10	0.0353 (15)	0.0350 (16)	0.0474 (16)	0.0028 (13)	0.0029 (12)	−0.0009 (14)
C11	0.0446 (18)	0.054 (2)	0.071 (2)	0.0005 (16)	0.0005 (16)	0.0201 (18)
C12	0.049 (2)	0.067 (2)	0.071 (2)	0.0112 (18)	−0.0079 (17)	0.0168 (19)
C13	0.0350 (16)	0.067 (2)	0.064 (2)	0.0000 (16)	−0.0008 (15)	0.0002 (19)
C14	0.0405 (18)	0.054 (2)	0.069 (2)	−0.0080 (16)	0.0076 (15)	0.0077 (18)
C15	0.0416 (17)	0.0445 (18)	0.0568 (18)	0.0030 (15)	0.0028 (14)	0.0060 (16)

### Geometric parameters (Å, º)

**Table:**

Br1—C1	1.910 (3)	C7—C8	1.370 (5)
O1—C9	1.353 (4)	C7—H7	0.9300
O1—C5	1.411 (3)	C8—H8	0.9300
O2—C2	1.198 (3)	C9—C10	1.485 (4)
O3—C9	1.191 (4)	C10—C15	1.372 (4)
C1—C2	1.503 (4)	C10—C11	1.384 (4)
C1—H1A	0.9700	C11—C12	1.378 (5)
C1—H1B	0.9700	C11—H11	0.9300
C2—C3	1.489 (4)	C12—C13	1.362 (5)
C3—C8	1.388 (4)	C12—H12	0.9300
C3—C4	1.395 (4)	C13—C14	1.359 (5)
C4—C5	1.363 (4)	C13—H13	0.9300
C4—H4	0.9300	C14—C15	1.387 (4)
C5—C6	1.372 (4)	C14—H14	0.9300
C6—C7	1.390 (4)	C15—H15	0.9300
C6—H6	0.9300		
			
C9—O1—C5	116.0 (2)	C7—C8—C3	121.0 (3)
C2—C1—Br1	114.3 (2)	C7—C8—H8	119.5
C2—C1—H1A	108.7	C3—C8—H8	119.5
Br1—C1—H1A	108.7	O3—C9—O1	122.3 (3)
C2—C1—H1B	108.7	O3—C9—C10	125.8 (3)
Br1—C1—H1B	108.7	O1—C9—C10	111.9 (3)
H1A—C1—H1B	107.6	C15—C10—C11	119.6 (3)
O2—C2—C3	121.6 (3)	C15—C10—C9	122.2 (3)
O2—C2—C1	122.6 (3)	C11—C10—C9	118.2 (3)
C3—C2—C1	115.8 (2)	C12—C11—C10	119.8 (3)
C8—C3—C4	118.5 (3)	C12—C11—H11	120.1
C8—C3—C2	119.3 (3)	C10—C11—H11	120.1
C4—C3—C2	122.2 (3)	C13—C12—C11	120.4 (3)
C5—C4—C3	119.6 (3)	C13—C12—H12	119.8
C5—C4—H4	120.2	C11—C12—H12	119.8
C3—C4—H4	120.2	C14—C13—C12	120.1 (3)
C4—C5—C6	122.4 (3)	C14—C13—H13	120.0
C4—C5—O1	117.6 (3)	C12—C13—H13	120.0
C6—C5—O1	120.0 (3)	C13—C14—C15	120.5 (3)
C5—C6—C7	118.3 (3)	C13—C14—H14	119.7
C5—C6—H6	120.9	C15—C14—H14	119.7
C7—C6—H6	120.9	C10—C15—C14	119.6 (3)
C8—C7—C6	120.3 (3)	C10—C15—H15	120.2
C8—C7—H7	119.8	C14—C15—H15	120.2
C6—C7—H7	119.8		
			
Br1—C1—C2—O2	2.7 (5)	C4—C3—C8—C7	−0.6 (4)
Br1—C1—C2—C3	−178.1 (2)	C2—C3—C8—C7	178.2 (3)
O2—C2—C3—C8	9.9 (5)	C5—O1—C9—O3	−7.3 (5)
C1—C2—C3—C8	−169.3 (3)	C5—O1—C9—C10	171.8 (2)
O2—C2—C3—C4	−171.4 (3)	O3—C9—C10—C15	168.8 (3)
C1—C2—C3—C4	9.4 (4)	O1—C9—C10—C15	−10.3 (4)
C8—C3—C4—C5	1.1 (4)	O3—C9—C10—C11	−10.1 (5)
C2—C3—C4—C5	−177.6 (3)	O1—C9—C10—C11	170.8 (3)
C3—C4—C5—C6	−0.8 (4)	C15—C10—C11—C12	1.0 (5)
C3—C4—C5—O1	−177.6 (2)	C9—C10—C11—C12	179.9 (3)
C9—O1—C5—C4	−95.2 (3)	C10—C11—C12—C13	−1.6 (6)
C9—O1—C5—C6	87.9 (3)	C11—C12—C13—C14	1.1 (6)
C4—C5—C6—C7	−0.1 (5)	C12—C13—C14—C15	0.1 (6)
O1—C5—C6—C7	176.6 (3)	C11—C10—C15—C14	0.2 (5)
C5—C6—C7—C8	0.7 (5)	C9—C10—C15—C14	−178.7 (3)
C6—C7—C8—C3	−0.3 (5)	C13—C14—C15—C10	−0.7 (5)

### Hydrogen-bond geometry (Å, º)

Cg2 is the centroid of the C10–C15 benzene ring.

**Table:**

*D*—H···*A*	*D*—H	H···*A*	*D*···*A*	*D*—H···*A*
C4—H4···O3^i^	0.93	2.70	3.557 (4)	153
C1—H1*B*···O3^i^	0.97	2.65	3.550 (4)	155
C6—H6···*Cg*2^ii^	0.93	2.88	3.627 (3)	138

Symmetry codes: (i) *x*, *y*−1, *z*; (ii) −*x*+1, −*y*+1, −*z*.
